# Ipilimumab in patients with melanoma and autoimmune disease

**DOI:** 10.1186/s40425-014-0035-z

**Published:** 2014-10-14

**Authors:** Chrisann Kyi, Richard D Carvajal, Jedd D Wolchok, Michael A Postow

**Affiliations:** New York-Presbyterian Hospital - Weill Cornell Medical College, 525 E 68th Street, New York, NY 10065 USA; Melanoma and Immunotherapeutics Oncology Service, Memorial Sloan Kettering Cancer Center, 300 East 66th Street, New York, NY 10065 USA; Weill Cornell Medical College, 525 E 68th Street, New York, NY 10065 USA

**Keywords:** Immunotherapy, Ipilimumab, Immune-related adverse events (irAEs), Autoimmune diseases, Rheumatoid arthritis, Multiple sclerosis, Melanoma

## Abstract

Cytotoxic T-Lymphocyte Antigen 4 (CTLA-4), an immune-checkpoint receptor and regulator of T-cell activation, has become an important therapeutic target for immunotherapy in cancer and autoimmune diseases. To date, clinical trials involving cancer immunotherapies, such checkpoint blocking antibodies directed at CTLA-4 (ipilimumab), have excluded patients with underlying autoimmune disease given concern for potential triggering of autoimmune exacerbations. We present the first known cases to our knowledge of two patients with active autoimmune diseases who received ipilimumab. In this limited clinical experience, no serious exacerbations of underlying autoimmunity have yet been observed, and one patient benefited from ipilimumab. These cases advocate for further investigation of the safety of cancer immunotherapies in cancer patients with underlying autoimmune conditions in carefully designed clinical trials with cautious monitoring.

## Background

Cytotoxic T-lymphocyte antigen 4 (CTLA-4), an immune-checkpoint receptor and regulator of T-cell activation, has become an important therapeutic target for immunotherapy in cancer. Ipilimumab, a fully human antibody that blocks CTLA-4, was the first immunomodulatory checkpoint inhibitor approved by the United States Food and Drug Administration (FDA) for patients with advanced melanoma [1,2]. Treatment with ipilimumab can be associated with inflammatory side effects, termed “immune-related adverse events” (irAEs) [3,4].

Given the critical role of CTLA-4 in maintaining immunologic homeostasis, clinical trials involving ipilimumab and cancer immunotherapies in general have excluded patients with underlying autoimmune diseases out of concern for triggering autoimmune exacerbations in these individuals. In preclinical models, anti-CTLA-4 treatment is known to enhance onset and severity of several T cell-mediated experimental autoimmune diseases, including murine models of encephalomyelitis [5,6], myasthenia gravis [7], and type 1 diabetes [8]. Yet, the clinical experience treating patients with ipilimumab who have advanced melanoma and concomitant, underlying autoimmune diseases has not been described.

We report two patients with advanced melanoma and a concomitant autoimmune disorder who were treated with ipilimumab; one had multiple sclerosis and another had rheumatoid arthritis. Ipilimumab was well tolerated in both patients without related exacerbation of their known autoimmune disease. One patient had a profound anti-tumor effect from ipilimumab.

## Case presentation 1

A 52-year-old man was diagnosed in March 2010 with multiple sclerosis (MS) when he presented to a neurologist with episodes of fatigue, lower extremity paresthesias, and bowel and bladder incontinence. The diagnosis of MS was made based upon the McDonald criteria with multiple clinical events associated with characteristic imaging findings [9]. He received initial treatment with glatiramer 20 mg daily via subcutaneous injection (an immunomodulatory drug that activates regulatory T-lymphocyte suppressor cells) and dalfampridine 20 mg twice daily orally (a potassium channel blocker affecting nerve conduction). After a year of treatment, he then received interferon beta 44mcg three times weekly via subcutaneous injection. His MS, characterized as the relapsing-remitting subtype, was active as he had suffered flares associated with increased weakness once every few months, requiring hospitalization and subsequent rehabilitation. Nonetheless, he was fully ambulatory and independent with his activities of daily living.

The patient’s melanoma history began shortly after initiation of treatment for MS when the patient first noted a lump on his right lower back. Shave biopsy revealed invasive, ulcerated melanoma to a depth of at least 3.5 mm Breslow thickness. He then underwent a wide excision and sentinel lymph node biopsy. Sentinel lymph node biopsy revealed micrometastatic disease in one lymph node in the right axilla, and a separate lymph node with melanoma was involved in the right inguinal region. Molecular analysis showed no detectable BRAF V600 mutation. After extensive discussion of options, the patient elected a program of vigilant observation rather than complete lymph node dissection of the two involved lymph node basins. Interferon alpha was discussed, but due to the patient’s active multiple sclerosis, he was thought not to be a good candidate for aggressive adjuvant therapy.

Nine months after wide excision, patient’s cancer recurred as multiple skin nodules involving his right flank, as well as right groin and right axilla lymphadenopathy. Excisional biopsy of a right groin nodule revealed metastatic melanoma. He was treated initially with temozolomide 75 mg/m2 daily in an extended dosing regimen [10]. After one month, a computed tomography (CT) scan showed evidence of disease progression with worsening lymphadenopathy, as well as progressive subcutaneous lesions on his right flank.

Due to limited treatment options and after carefully considering the risks and benefits of treatment, standard ipilimumab was initiated at 3 mg/kg every three weeks for four doses. During ipilimumab, he had no significant irAEs and no exacerbation of his MS. He continued to receive interferon beta treatment for his multiple sclerosis while receiving ipilimumab. Unfortunately, after completing ipilimumab, a positron emission tomography (PET) scan showed progressive disease with worsening widespread metastatic melanoma. Despite subsequent treatment with carboplatin and paclitaxel, he died due to melanoma progression in March 2013.

## Case presentation 2

A 67-year-old woman presented to a rheumatologist with anemia, weight loss, and multiple joint effusions. Based upon clinical criteria as per the American College of Rheumatology/European League Against Rheumatism collaborative initiative [11], she was diagnosed with seronegative rheumatoid arthritis (RA) in 2007. She underwent a left knee aspiration, showing 16,000 white blood cells with neutrophil predominance. She was then started on treatment with prednisone 20 mg daily and methotrexate 15 mg weekly with significant improvement in her symptoms.

Around this time, she was also diagnosed with cutaneous melanoma. She first noticed a pigmented lesion on her right calf in 2005. Biopsy of the right leg lesion was performed, with pathology revealing invasive melanoma, 0.72 mm in Breslow thickness, no ulceration was seen. Local excision of the melanoma and right inguinal sentinel lymph node biopsy one month later showed no residual malignant melanoma.

Following initial surgical resection, the patient remained without melanoma recurrence until November 2011 when she noticed subcutaneous lesions in the left anterior neck and right lower back. She underwent skin excisional biopsy of the suspicious site on her right lower back with pathology consistent with metastatic melanoma. Molecular analysis revealed no apparent mutation in BRAF, NRAS or KIT. Whole body PET/CT scan revealed metastatic disease with multiple lung and subcutaneous nodules.

Though ipilimumab was initially considered, it was deferred out of concern it would exacerbate the patient’s RA, which still required methotrexate and low-dose corticosteroids for maintenance. Instead, the patient was treated on a clinical trial involving a gamma secretase inhibitor in combination with cisplatin, vinblastine, and temozolomide (CVT) chemotherapy. Despite initial clinical benefit with this regimen, melanoma progression was observed eight months later. Given limited treatment options, ipilimumab was pursued after a complete discussion of its potential risks and benefits in the setting of known RA.

**Figure 1:**
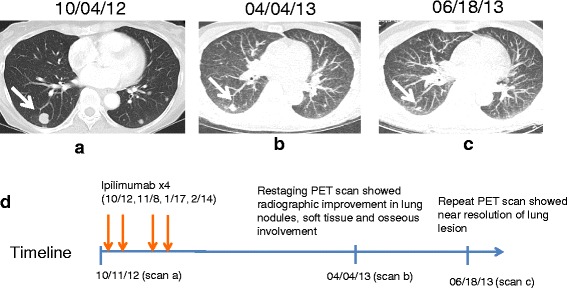
**Timeline of treatment and demonstration of response. (a)** Baseline chest CT scan prior to ipilimumab showed multiple subcutaneous, intramuscular, and pulmonary nodules (largest nodule indicated by white arrow). **(b)** Two months after completion of four treatments with ipilimumab, restaging PET scan showed decreased size of lung nodules, soft tissue and osseous involvement. **(c)** Repeat CT scan showed near radiographic resolution of lung nodule. **(d)** Timeline of described events (not to scale).

In October 2012, the patient was initiated on a course of ipilimumab (3 mg/kg every three weeks for four doses). After completing four doses of ipilimumab, subsequent imaging showed partial response, with overall decreased bilateral lung nodules, soft tissue and osseous involvement (Figure 1). Following completion of ipilimumab, she did note slightly increased bilateral knee pain consistent with known osteoarthritis rather than RA, and this was effectively treated with celecoxib. Throughout this course, the patient was maintained on methotrexate 15 mg weekly and low-dose prednisone (5 mg daily).

In January 2014, over one year since starting ipilimumab, a computed tomography (CT) scan demonstrated slow progression of disease. Given the long initial response, ipilimumab re-induction has been pursued, and she is currently undergoing this treatment. She has had no worsening symptoms related to RA throughout the remainder of course to date.

## Conclusions

We describe two patients with active autoimmune diseases treated with ipilimumab for metastatic melanoma due to limited alternative treatment options. To our knowledge, this is the first description of patients with active autoimmune diseases to have received ipilimumab. Despite the known irAE profile of ipilimumab and the theoretical concern that ipilimumab could exacerbate an underlying autoimmune disorder, whether ipilimumab truly exacerbates underlying autoimmune conditions remains unknown.

The patient described in case 2 had benefit from ipilimumab. In this patient, the ongoing immune suppression (corticosteroids and methotrexate) required to optimally treat RA, did not preclude a response to ipilimumab. This finding is consistent with other recently published cases where patients benefited from ipilimumab, despite ongoing low-dose immunosuppression needed for prior solid organ transplants [12]. There remains no published literature documenting that concurrent immunosuppressive treatments eliminate the possibility of a favorable therapeutic outcome from ipilimumab, and the potential benefit of ipilimumab in this context merits further study.

Notably, patient 1 was diagnosed with melanoma soon after starting immunosuppression for MS. Patient 2 experienced a melanoma recurrence after initiation of immunosuppression for RA. While initial immunosuppression and timing of melanoma may have been completely independent events and due to increased clinical contact, it is theoretically possible that they were related as has been previously described [13].

We recognize these are only two patient experiences, and anecdotal experiences cannot be seen as definitive evidence for the safety of immunotherapy in this setting. The experiences with these two patients’ autoimmune conditions may also not be generalizable to patients with other autoimmune disorders, which may have different underlying pathophysiology.

Nevertheless, these experiences advocate for further evaluation of cancer immunotherapies for patients with underlying autoimmune conditions. Professional societies and patient advocacy groups may consider mechanisms for patient registries, and carefully designed clinical trials with cautious monitoring will help to increase our understanding of immunotherapies in this patient population. This may become increasingly important as approaches targeting the programmed cell death-1 (PD-1) receptor, which is typically associated with minimal inflammatory toxicity, expand in multiple malignancies. Such efforts will help define the tenuous line between enhancing antitumor immunity and avoiding exacerbation of an underlying autoimmune disorder.

## Consent

Written informed consent was obtained from the patient or next of kin for publication of this case report and any accompanying images. Copies of the written consents are available for review by the Editor-in-Chief of this journal.

## Abbreviations

CTLA-4: Cytotoxic T-lymphocyte-associated antigen 4
irAE: Immune-related adverse event
MS: Multiple sclerosis
RA: Rheumatoid arthritis
